# Evaluation of the Nrf2-Keap1 Pathway in Patients with Acute Cerebral Ischemic Disease

**DOI:** 10.3390/medicina62071371

**Published:** 2026-07-16

**Authors:** Gizem Alkan, Fatih Koçtürk, Ayşe Karakus, Muhammed Enes Taysi, Seyithan Taysi

**Affiliations:** 1 Department of Medical Biochemistry, Faculty of Medicine, Gaziantep University, Gaziantep 27310, Turkey; gizemalkan44@gmail.com; 2 Department Neurology, Faculty of Medicine, Kahramanmaras Sutcu Imam University, Kahramanmaras 46050, Turkey; drfatihkocturk@gmail.com; 3 Department Neurology, Faculty of Medicine, Gaziantep University, Gaziantep 27310, Turkey; sirtlanayse08@gmail.com; 4 Division of Emergency Medicine, Cankiri State Hospital, Cankiri 18100, Turkey; enestaysi65@gmail.com; 5 Phytotherapy and Medicinal-Aromatic Plants Application and Research Center, Gaziantep University, Gaziantep 27310, Turkey

**Keywords:** acute cerebral ischemic disease, Nrf2–Keap1 signaling pathway, oxidative stress, lipid peroxidation, 4-hydroxynonenal, glutathione

## Abstract

*Background and Objectives*: Acute cerebral ischemia is characterized by excessive oxidative stress and impaired antioxidant defense mechanisms, in which the nuclear factor erythroid 2–related factor 2 (Nrf2)–Kelch-like ECH-associated protein 1 (Keap1) signaling pathway plays a pivotal regulatory role. This study aimed to investigate serum levels of Nrf2–Keap1 pathway components and associated oxidative stress biomarkers in patients with acute ischemic stroke. *Materials and Methods*: Eighty-eight patients diagnosed with ischemic stroke who presented within 24 h of the onset of neurological deficit and met the inclusion criteria, along with 72 healthy control subjects without a history of acute ischemic stroke, were included in the study. Serum levels of Nrf2, Keap1, glycogen synthase kinase-3β (GSK-3β), heme oxygenase-1 (HO-1), glutathione (GSH), and 4-hydroxynonenal (4-HNE) were quantified using enzyme-linked immunosorbent assay (ELISA). Receiver operating characteristic (ROC) analysis was performed to evaluate the diagnostic performance of the biomarkers. *Results*: Compared with controls, patients exhibited significantly reduced Nrf2 levels and markedly elevated Keap1 and 4-HNE levels. HO-1 and GSH concentrations were also significantly increased in the patient group, whereas GSK-3β levels did not differ significantly between groups. ROC analysis demonstrated that 4-HNE and Nrf2 possessed the highest discriminative capacity for acute ischemic stroke. *Conclusions*: These findings suggest that acute cerebral ischemia is associated with dysregulation of the Nrf2–Keap1 axis accompanied by enhanced lipid peroxidation and oxidative burden. Although increased HO-1 and GSH levels may reflect a compensatory antioxidant response, elevated 4-HNE levels indicate persistent oxidative injury.

## 1. Introduction

In living organisms, there is a balance between the rate of free radical formation and the rate at which they are eliminated from the system, and this balance is called oxidative balance (redox homeostasis). As long as this balance exists, the radical species formed in the organism can be neutralized; however, this balance is disrupted if radical production increases or the effectiveness of antioxidant defense systems decreases [1,2,3]. Free radicals, especially reactive oxygen species (ROS) [4] and reactive nitrogen species (RNT), react with macromolecules (e.g., lipids, proteins, DNA) to form various degrading products and alter the structure of these macromolecules [5,6,7]. This disrupted redox balance plays an important role in the pathogenesis of chronic diseases, especially neurodegenerative diseases, chronic inflammatory processes, cardiovascular diseases, and metabolic diseases [4,8,9].

One of the most important protective response pathways against damage caused by oxidative stress in living cells occurs via nuclear factor erythroid 2-related factor 2 (Nrf2). Nrf2 is one of the main antioxidant transcription factors responsible for the synthesis of enzymes that both neutralize ROS and play a role in detoxification processes. In addition, Nrf2 regulates the gene expression of molecular chaperones involved in the cellular stress response, proteasome components, and various enzymes involved in intermediate metabolisms [10,11,12].

Under physiological conditions, Nrf2 exists inactively in the cytoplasm in a complex with Kelch-like ECH-associated protein 1 (Keap1), a protein that suppresses it. However, when the cell is exposed to oxidative stress, these complexes dissociate; Nrf2 is released, activated, and passes into the nucleus [10]. In the nucleus, it binds to gene regions containing antioxidant response elements (AREs) and stimulates the transcription of these genes. Thus, Nrf2 plays a protective role not only against the harmful effects of oxidative stress but also against inflammatory and apoptotic processes, contributing to the maintenance of cellular balance and homeostasis [8,10].

Stroke is a major public health problem, currently being one of the leading causes of death and disability worldwide. Affecting millions of people every year, this disease leads not only to loss of life but also to long-term physical disabilities [13]. Approximately half of individuals who experience a stroke develop permanent motor function loss, necessitating long-term rehabilitation processes [14]. Therefore, stroke not only reduces the quality of life but also places significant social and economic burdens on families and healthcare systems [15]. Economic analyses conducted across Europe show that the financial burden of stroke treatment and care processes on healthcare systems is quite high. A systematic review and meta-analysis by Capone et al. [16] reported that stroke treatment and care costs constituted approximately 4% of total healthcare expenditures. In contrast, a more recent population-based analysis by Lucas et al. [17] indicated that this rate was around 1.7%. Both studies highlight that with the aging population and increasing prevalence of chronic diseases in Europe, the indirect costs associated with stroke will increase further in the coming years [16,17,18].

Acute cerebral ischemia results from a sudden interruption of blood flow to a specific area of the brain, initiating a complex chain of events that can quickly lead to permanent nerve tissue damage. In this process, decreased energy production due to oxygen and glucose deficiency disrupts ion balance in the cell membrane and leads to intracellular calcium accumulation. Increased calcium levels trigger excessive glutamate release, mitochondrial dysfunction, and overproduction of ROS, leading to oxidative stress [19,20,21]. These biochemical events affect not only the cells in the infarct area but also the surrounding salvageable nerve tissue, known as the “penumbra” [22]. Therefore, limiting ischemic damage early and preventing secondary cell death are critical to the success of treatment [23,24].

Recent experimental and preclinical studies have clearly demonstrated that the Nrf2–Keap1 signaling pathway is one of the organism’s own protective mechanisms against ischemic brain events [12]. In particular, when the Nrf2/Keap1/ARE pathway is activated, neuronal cells become more resistant to damage caused by oxidative stress, inflammatory processes, and apoptotic signals, playing a key role in limiting damage to brain tissue [25]. In this context, molecules involved in the regulation of this signaling pathway or biomarkers reflecting its effects have begun to be considered as potential tools in understanding stroke pathophysiology and developing early intervention strategies [26]. Within the chain reactions that begin with oxygen–glucose deficiency following brain ischemia, the Nrf2–Keap1 system acts like an alarm mechanism. Nrf2, which is “on standby” in the cytoplasm with Keap1, is released when stress conditions arise, moves to the nucleus, and activates genes that trigger antioxidant response elements. In this way, intracellular DNA, lipid and protein damage can be reduced; at the same time, the inflammatory microenvironment is suppressed and pathways leading to cell death can be stopped early [27]. This holds great promise for improving neuroprotection and rehabilitation processes after stroke.

Research shows that glycogen synthase kinase 3 beta (GSK-3β) is actually an important actor not only in glycogen metabolism but also in cellular antioxidant defense systems. GSK-3β can suppress antioxidant responses by disrupting the translocation or stability of NFE2L2 (Nrf2) to the nucleus [28,29]. For example, when Nrf2 is activated, one of its target genes, HMOX1 (HO-1), is expressed at high levels. Heme oxygenase-1 (HO-1) acts as a defense protein with both antioxidant and anti-inflammatory effects, breaking down the heme molecule to produce biliverdin, carbon monoxide, and iron [30]. On the other hand, glutathione (GSH), a key component of intracellular redox balance, is protected thanks to gene expression directed by Nrf2, thus protecting cells under oxidative load [31].

At the same time, 4-hydroxynonenal (4-HNE), a highly reactive aldehyde formed as a result of lipid peroxidation, is considered not only a classic damage indicator but also a signaling molecule that can trigger the Nrf2/Keap1 system. At low levels, 4-HNE creates an alarm system in the cell; by disrupting Keap1-Nrf2 binding, it can mediate Nrf2 remaining in the nucleus for a longer period and the activation of antioxidant genes [32]. From this perspective, focusing on the GSK-3β-HO-1-GSH axis and lipid-derived signaling molecules such as 4-HNE has great potential for controlling oxidative stress and cellular damage. Consequently, when these three (GSK-3β, HO-1, and GSH) are considered together, they function as a “line of defense” at the cellular level: GSK-3β is a negative regulator, while HO-1 and GSH form the active defense layers. If this system is disrupted, the cell can become vulnerable to processes such as oxidative stress, inflammation, and apoptosis. Therefore, a thorough understanding of these pathways is becoming increasingly important as a therapeutic target in both neurological and systemic diseases.

This study aims to investigate serum levels of the Nrf2–Keap1 signaling pathway in patients with acute cerebral ischemic stroke, to examine its association with ischemic process-related biomarkers, and to elucidate its role in the oxidative stress response. Specifically, this study aims to evaluate the potential regulatory effects of GSK-3β levels on Nrf2 activity, determine the levels of antioxidant molecules regulated by Nrf2, such as HO-1 and GSH, and analyze 4-HNE, a toxic product of lipid peroxidation, in terms of both its role as an oxidative stress indicator and its potential interactions with Nrf2. In light of this information, we aimed to investigate the role of the Nrf2–Keap1 axis in stroke pathophysiology and to explore this pathway as a potential diagnostic and therapeutic target.

## 2. Methods

### 2.1. Ethical Approval

This study was approved by the Clinical Research Ethics Committee of Gaziantep University on 14 February 2024, with decision number 2024/15. Before enrollment, all participants were informed in detail about the study protocol, and written informed consent was obtained from each participant.

### 2.2. Participants

A total of 88 patients who were examined by a neurologist, diagnosed with ischemic stroke, evaluated within the first 24 h following the onset of neurological deficits, and met the inclusion criteria were enrolled in the study. In addition, 72 healthy individuals without a history of acute ischemic stroke were included as the control group. Patients aged between 18 and 80 years who were diagnosed with acute ischemic stroke within the first 24 h of symptom onset were included in the study. Pregnant women, breastfeeding mothers, and patients with hemorrhagic stroke were excluded from the study.

### 2.3. Measurement of Biochemical Parameters

10 mL of venous blood sample was collected from each participant in anticoagulant-free tubes. After the collected blood samples were kept at room temperature for approximately 30 min, they were centrifuged at 4000× *g* for 5 min. The obtained sera were stored in Eppendorf tubes at −80 °C until analysis.

In this study, the levels of Nrf2, Keap1, GSK-3β, HO-1, GSH, and 4-HNE were examined in serum samples obtained from patients. Measurements were performed using an enzyme-linked immunosorbent assay (ELISA) specific to human proteins. All analyses were performed using commercial kits manufactured by Bioassay Technology Laboratory (BT Lab, Shanghai, China), according to the manufacturer’s recommended protocols. The catalog numbers of the kits used were recorded as follows: E3244Hu for Nrf2, E5534Hu for Keap1, E3196Hu for GSK-3β, E0932Hu for HO-1, EA0142Hu for GSH, and EA0066Hu for 4-HNE.

Before initiating the analyses, all reagents, standard solutions, and serum samples were equilibrated by bringing them to room temperature. For each test, standard curves were generated using the standard solutions provided with the kits. Specified volumes (approximately 40–50 µL) of serum samples were transferred to the wells on the plate. Then, biotin-labeled antibodies and streptavidin-HRP conjugates were added. The mixtures were incubated at 37 °C for approximately one hour. Following incubation, the plates were washed five times with a washing buffer containing PBS to remove free reagents. Subsequently, Substrate Solutions A and B were added to each well, respectively, and the plates were incubated at 37 °C for 10 min in a light-free environment. After the color reaction was complete, the blue color turned yellow when 50 µL of stop solution was added. This color change indicated that the reaction was successfully completed. In the final step, the color intensity in each well was measured using a microplate reader (Thermo Fisher Scientific, Waltham, MA, USA). Results were expressed in ng/mL for Nrf2, HO-1 and GSH, and in ng/L for Keap1, GSK-3β and 4-HNE.

## 3. Statistical Analyses

The data obtained in this study were evaluated comparatively between patients with acute ischemic stroke and a healthy control group. The distribution of continuous variables was examined using the Kolmogorov–Smirnov test, and since the data did not show normal distribution, the Mann–Whitney U test was used for comparisons between groups. Findings were presented as mean ± standard deviation (SD). The optimum cutoff value for all variables was determined using ROC analysis. All statistical analyses were performed using the SPSS 27.0 (IBM Corp., Armonk, NY, USA) program, and a *p* < 0.05 value was considered significant.

Additionally, we used MetaboAnalyst 6.0 software to examine the differences in biomarkers between the two groups at the multivariate level. To control for potential multiple-comparison bias arising from the simultaneous evaluation of multiple biomarkers, false discovery rate-adjusted values automatically generated by MetaboAnalyst 6.0 were also taken into consideration. The FDR-adjusted results were reviewed, and the biomarkers reported as statistically significant in the manuscript remained significant after FDR correction.

Multivariate statistical analyses were performed using MetaboAnalyst. Before PCA and PLS-DA analyses, the dataset was checked for missing values. Since the variables were measured as absolute concentration values, no additional sample normalization was applied. To reduce data skewness and minimize the influence of different measurement ranges, the data were log-transformed and Pareto-scaled prior to multivariate analysis. PCA was used as an unsupervised method to explore the overall variance structure of the dataset and to visualize the natural distribution of acute ischemic stroke patients and healthy controls. PLS-DA was performed as a supervised method to evaluate group separation and to determine the variables contributing most strongly to the discrimination between the groups.

## 4. Results

Our statistical analyses revealed some significant biochemical differences between the patient and control groups. Specifically, the significantly lower levels of Nrf2 (*p* < 0.001) and higher levels of Keap1 (*p* < 0.001) in the patient group indicate suppressed antioxidant responses. Significant changes in HO-1 and GSH levels suggest the body is attempting to mount a response to oxidative stress.

One of the most striking findings was the significantly higher levels of 4-HNE in the patient group (*p* < 0.001), indicating increased lipid peroxidation. GSK-3β levels, however, did not show a significant difference between the two groups (*p* = 0.952). In general, the results indicate increased oxidative stress in the patient group and that the relevant molecular mechanisms are regulated differently than in the control group.

**Figure 1:**
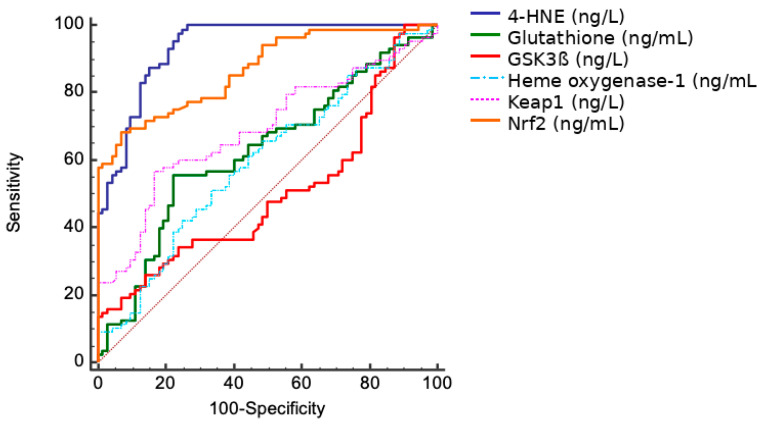
ROC analysis for all biochemical variables.

ROC analysis clearly demonstrated the diagnostic power of the biomarkers in the study to differentiate the patient group from the control group. Examining the graph, it is particularly noticeable that the 4-HNE curve (dark blue) deviates the most from the reference line and rises most significantly to the left. This behavior suggests that 4-HNE operates with high sensitivity and specificity and exhibits strong diagnostic performance. Similarly, Nrf2 (orange curve) also shows a very successful performance; the more stable upward progression of its curve supports the possibility that this parameter may be valuable in differentiating the patient group (Figure 1).

In addition, we see that the GSH, Keap1, and HO-1 curves deviate significantly from the reference line, meaning these parameters also have a diagnostic contribution. However, looking at the overall slope of the curves, they do not seem to be as strong as 4-HNE and Nrf2. GSK-3β, on the other hand, follows a course closer to the reference line compared to the others, suggesting that it does not show a very significant discriminatory effect from a diagnostic point of view. Looking at the overall picture, we can say that 4-HNE stands out as the strongest biomarker, followed by Nrf2, and the other parameters offer moderate diagnostic value.

**Figure 2:**
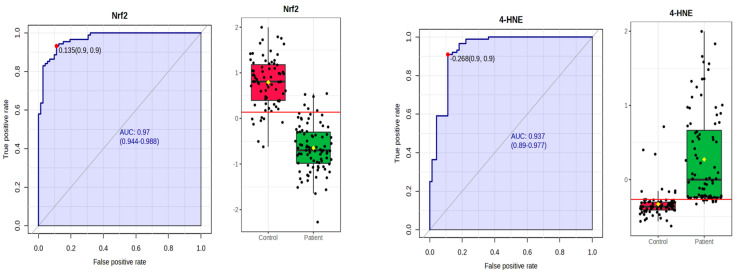
ROC analysis of NRF-2 and 4 HNE values. Evaluation of ROC analysis.

Figure 2 shows that the AUC value for Nrf2 is quite high (AUC = 0.970; 95% CI: 0.944–0.988). This suggests that Nrf2 demonstrates a strong distinguishing performance in differentiating the patient group from the control group. The boxplot also supports this differentiation, showing higher Nrf2 levels in the control group and lower levels in the patient group. The optimal cutoff value for 4-HNE was −0.285, with 90% sensitivity and 90% specificity. In other words, both the ROC curve and the boxplot suggest that 4-HNE may serve as a biomarker indicating the presence of the disease.

**Figure 3:**
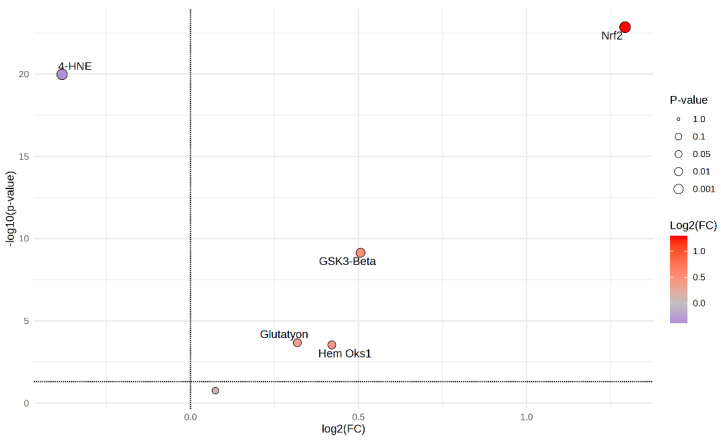
Log2 fold-change levels of oxidative stress-related parameters.

Figure 3 presents the log2-fold change values of the evaluated biomarkers between the patient and control groups. The horizontal black line indicates a log2FC = 0 level. Positive values above this line indicate parameters that are at higher levels in the patient group compared to the control group, while negative values below the line indicate parameters that are at lower levels in the patient group. The most significant positive change in the graph was observed in 4-HNE. Keap1 also showed a significant increase in the log2FC value in the patient group. HO-1 and glutathione parameters were also found to be at higher levels in the patient group with positive log2FC values; however, the magnitude of these changes was more limited compared to 4-HNE and Keap1. In contrast, Nrf2 was the only parameter observed to be at a lower level in the patient group compared to the control group with a negative log2FC value. Overall, this graph visually supports the group comparisons given in Table 1. 4-HNE, Keap1, HO-1, and glutathione are higher in the patient group; Nrf2 is lower in the patient group. In GSK-3β, no significant fold change was observed, which is consistent with the lack of statistically significant differences between the groups shown in Table 1.

**Figure 4:**
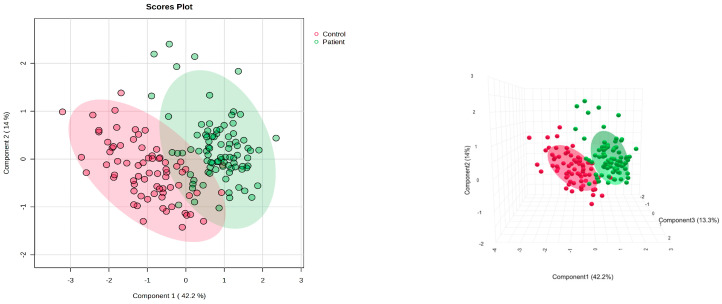
Two-Dimensional and Three-Dimensional Distribution of Groups.

Figure 4 shows the two-dimensional and three-dimensional score distribution graphs for the patient and control groups. Red dots represent the control group, and green dots represent the patient group. Each dot shows the overall profile of a participant based on the measured biomarkers. In the two-dimensional score graph, the first component explains 42.2% of the total variation, and the second component explains 21.5%. When these two components are considered together, they provide a general visual distinction in the distribution of samples belonging to the patient and control groups. In the three-dimensional score graph, the distribution of the groups is shown in more detail with the addition of the third component, which represents 13.3% of the total variation. Colored ellipses show the general distribution areas of the groups. Although there is partial overlap between the groups in the graphs, it is observed that the patient and control samples show a tendency to be distributed in different regions. This visual distribution shows that the groups differ in terms of their multivariate biomarker profiles.

**Figure 5:**
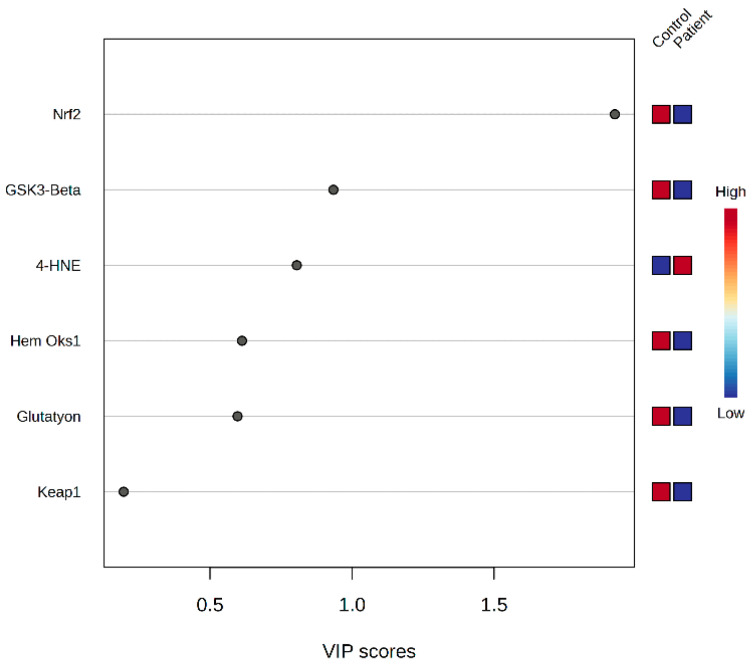
VIP Score.

Figure 5 shows the VIP scores obtained from the PLS-DA model. The VIP score indicates the relative contribution of each biomarker to explaining the multivariate distinction between patient and control groups. In general, a VIP score above 1 indicates that the variable in question contributes more significantly to group differentiation in the model.

In this graph, the variables with VIP scores above 1 are identified as 4-HNE and Keap1. 4-HNE is the parameter with the highest VIP score and was observed to be the variable that most strongly contributed to the differentiation between patient and control groups. Keap1 also showed a significant differentiating contribution in the model as the other parameter with a VIP score above 1.

The color boxes on the right side of the graph show the relative level of each biomarker in the control and patient groups. In the boxes, red indicates a higher relative level, and blue indicates a lower relative level. According to this color distribution, 4-HNE and Keap1 are at higher levels in the patient group and lower levels in the control group. The opposite is observed in Nrf2; Higher relative levels were observed in the control group and lower levels in the patient group. Since the VIP scores of GSK-3β, Nrf2, glutathione, and HO-1 parameters remained below 1, these variables were not considered among the parameters that primarily contribute to group differentiation in the PLS-DA model.

**Figure 6:**
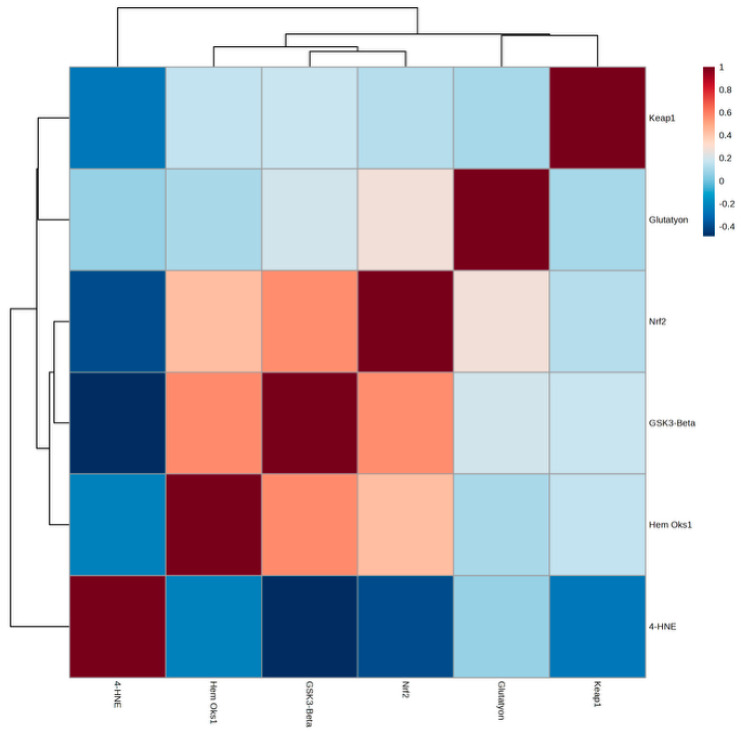
Correlation Map.

Figure 6 presents the correlation relationships between the evaluated biomarkers in the form of a heat map. In the map, red tones indicate positive correlation, and blue tones indicate negative correlation. As the color intensity increases, the strength of the correlation also increases. In addition, the dendrograms located at the top and left show the clustering of parameters showing similar correlation characteristics. While positive correlation trends are observed between some biomarkers in the graph, negative correlation trends are noticeable between some parameters. In particular, 4-HNE shows an inverse correlation relationship with Nrf2, GSK-3β, and HO-1. In contrast, more pronounced positive correlation trends are observed between Nrf2, GSK-3β, and HO-1. Keap1 and glutathione show varying levels of correlation with different parameters. The correlation map visually summarizes the relationships between the measured biomarkers and shows that the correlation patterns between the parameters are not homogeneous. This analysis complements group comparisons and shows the direction and relative strength of the relationships between variables.

## 5. Discussion

The findings of this study reveal a significant imbalance in key regulators of the Nrf2 signaling pathway and indicators of oxidative stress in the patient group. The significantly higher plasma levels of Keap1 and 4-HNE, together with the significantly lower levels of Nrf2 in the patient group compared with the control group, are consistent with an altered oxidative stress response and are associated with changes in antioxidant defense-related pathways. The increase in 4-HNE, a strong indicator of lipid peroxidation, suggests that oxidative damage is more pronounced at the systemic level. In contrast, the significant increase in HO-1 and GSH levels indicates the involvement of compensatory antioxidant responses at the cellular level. The lack of a significant difference in GSK-3β levels between the groups suggests that this molecule may play a secondary or indirect role in this pathological process. Overall, these results demonstrate an increased oxidative stress load during disease, impaired regulation of the Nrf2-Keap1 axis, and an inability of accompanying antioxidant responses to maintain adequate balance, supporting the idea that components of this pathway may be critically important in pathophysiological processes. In general, these results demonstrate an increase in oxidative stress during disease, impaired regulation of the Nrf2-Keap1 axis, and an inability of accompanying antioxidant responses to maintain adequate balance, supporting the hypothesis that components of this pathway may be critically important in pathophysiological processes.

Acute cerebral ischemic stroke is a complex neurovascular disease resulting from a sudden and critical decrease in cerebral blood flow, characterized by high mortality and morbidity [33]. The reperfusion process following ischemia creates a significant oxidative stress environment characterized by excessive production of ROS, lipid peroxidation, mitochondrial dysfunction, and deficiency of cellular antioxidant defense systems [34]. In this process, oxidative stress is considered one of the fundamental pathogenic mechanisms in the progression of neuronal damage and the development of secondary brain damage [35,36]. The Nrf2-Keap1 signaling pathway, one of the main defense mechanisms developed by cells against oxidative stress, activates antioxidant response elements and regulates the expression of HO-1, glutathione metabolism, and phase II detoxification enzymes [37,38]. Experimental and clinical studies have shown that Nrf2 activation during cerebral ischemia supports neuronal survival, limits the inflammatory response, and reduces cellular damage due to lipid peroxidation [39]. In contrast, it has been reported that continued Keap1-mediated Nrf2 suppression deepens oxidative damage and increases cell death in ischemic brain tissue [40]. These findings suggest that the Nrf2-Keap1 axis plays a central role in maintaining redox balance in acute cerebral ischemia and that this pathway could be considered among potential diagnostic and therapeutic targets [12,41].

Recent studies have revealed that inflammation and oxidative stress are intertwined fundamental mechanisms in the early and late pathophysiology of acute cerebral ischemia [42]. Pro-inflammatory cytokines released by microglia and endothelial cells activated after an ischemic event disrupt the integrity of the blood–brain barrier, deepening the neuroinflammatory response and increasing neuronal damage [43]. Simultaneously with this inflammatory process, increased ROS lead to cellular disorders such as lipid peroxidation, protein oxidation, and DNA damage in cerebral tissue, significantly increasing the oxidative stress burden [44]. In acute cerebral ischemia, the severity of oxidative stress is further aggravated by the deficiency of antioxidant defense systems, leading to a disruption of cellular redox balance [45]. Clinical and experimental studies show that antioxidant capacity is decreased in patients with ischemic stroke, while oxidant markers and lipid peroxidation products are significantly increased [46]. For this reason, the bidirectional interaction between inflammation and oxidative stress plays a critical role in shaping secondary brain damage in acute cerebral ischemia, further highlighting the importance of redox-sensitive signaling pathways [47].

Nrf2 plays a central role in maintaining cellular redox balance, regulating the levels of ROS/RNS through multiple mechanisms [48]. Activated during ischemic brain injury, Nrf2 binds to antioxidant response elements, increasing the expression of numerous cytoprotective genes such as HO-1, glutathione biosynthesis enzymes, and phase II detoxification systems [49]. Through this regulatory effect, Nrf2 contributes to the maintenance of cellular homeostasis under oxidative stress conditions and supports the survival capacity of neuronal cells [50]. Furthermore, the Nrf2 signaling pathway has been shown to influence multiple cellular processes such as autophagy activation, inflammasome suppression, modulation of the endoplasmic reticulum stress response, and maintenance of mitochondrial function [51]. In the context of acute cerebral ischemia, these mechanisms are critical in limiting secondary brain damage and slowing the progression of ischemic tissue damage [52]. Therefore, it is thought that activating Nrf2 through endogenous or pharmacological pathways may provide neuroprotective effects against oxidative and inflammatory damage and constitute a promising target for new treatment strategies for cerebral ischemia [53].

The decrease in Nrf2 levels together with the increase in Keap1 levels observed in the patient group is associated with alterations in the Nrf2–Keap1 signaling pathway. It may reflect changes in antioxidant defense-related mechanisms during acute cerebral ischemia [54]. It should also be considered that circulating Nrf2 and Keap1 concentrations may not directly reflect intracellular signaling activity. Rather, serum levels are thought to represent a combination of passive release from injured or dying cells, extracellular vesicle/exosomal release, and other systemic responses to tissue injury. Consequently, circulating concentrations are more appropriately interpreted as systemic biochemical indicators associated with oxidative stress than as direct measures of intracellular Nrf2–Keap1 pathway activity within ischemic brain tissue. This distinction should be considered when interpreting the biological significance of serum biomarker alterations observed in the present study [55,56]. It has been reported that the increase in Keap1 expression restricts the nuclear translocation of Nrf2, thus inhibiting the activation of antioxidant genes and contributing to the disruption of redox balance [57,58]. The decrease in Nrf2 levels is thought to be related to the inadequacy of cellular defense mechanisms in the face of increased oxidative load under ischemic conditions [59]. This molecular imbalance is consistent with the significant increase in 4-HNE levels, a strong indicator of lipid peroxidation detected in our study, showing that oxidative damage is increased at the systemic level [60]. In contrast, the increase in HO-1 and GSH levels suggests that cells are trying to develop a compensatory response to oxidative stress, but this response is not sufficient to balance the increased oxidative load [61]. Taken together, it appears that the regulation disorder in the Nrf2-Keap1 signaling pathway plays a critical role in the formation and maintenance of oxidant/antioxidant imbalance in acute cerebral ischemia [62,63]. Studies show that lipid peroxidation is one of the key determinants of oxidative damage in acute cerebral ischemia and is closely associated with neuronal cell death [64,65].

The excessive production of ROS during ischemia–reperfusion promotes lipid peroxidation of membrane polyunsaturated fatty acids, thereby generating biologically active reactive aldehydes [45]. Among these aldehydes, 4-HNE is one of the most toxic end products of lipid peroxidation, disrupting cellular signaling and metabolic balance by forming covalent bonds with proteins, lipids, and nucleic acids [66]. Clinical and experimental data reveal that serum 4-HNE levels are significantly increased in patients with acute ischemic stroke, and this increase correlates with the severity of ischemic damage [67,68]. Elevated 4-HNE levels are reported to impair the integrity of the neurovascular unit through endothelial dysfunction, mitochondrial damage, and activation of pro-inflammatory pathways [69,70]. The high 4-HNE levels detected in the patient group in our study can be considered as a systemic reflection of increased oxidative stress and lipid peroxidation in acute cerebral ischemia and support the pathophysiological importance of oxidative damage [45].

Beyond its diagnostic relevance, the observed decrease in Nrf2 together with increased Keap1 and 4-HNE levels may have important clinical implications. The Nrf2–Keap1 pathway is increasingly recognized as a therapeutic target in ischemic stroke because restoration of Nrf2 activity has been shown to attenuate oxidative stress, neuroinflammation, and ischemia–reperfusion injury by enhancing endogenous antioxidant defenses. In addition, impaired Nrf2 signaling has been implicated in ferroptosis, a lipid peroxidation-driven form of regulated cell death that contributes to neuronal injury after cerebral ischemia. Although our study does not establish mechanistic or causal relationships, the observed biomarker profile supports the hypothesis that dysregulation of the Nrf2–Keap1 axis may contribute to these pathological processes. Further mechanistic and prospective clinical studies are warranted to determine whether targeting this pathway could provide therapeutic benefit in ischemic stroke [71].

The high AUC values obtained in this study, especially for 4-HNE and Nrf2, may be effective in distinguishing between patient and control groups of these biomarkers. However, these results should be interpreted cautiously due to the lack of an independent validation cohort. The AUC values obtained from the ROC analysis reflect the discriminatory performance within the current study population and do not directly imply clinical diagnostic validity. Therefore, further studies with larger sample sizes, multicenters, and independent validation cohorts are needed to confirm the diagnostic value of these biomarkers.

Glycogen synthase kinase-3β is a key serine/threonine kinase involved in the regulation of inflammation, energy metabolism, apoptosis, and cellular stress responses, and is considered an important regulator in the pathogenesis of neurological diseases [72]. It has been reported that GSK-3β activation in acute cerebral ischemia may be associated with enhanced pro-inflammatory signaling pathways, increased mitochondrial dysfunction, and increased neuronal cell death [73]. However, it is also emphasized that the role of GSK-3β in ischemic processes can vary depending on time, tissue, and cellular context, and does not show a consistent increase in all clinical studies [74]. The lack of a significant difference in GSK-3β levels between patient and control groups in our study suggests that this kinase may not show a significant change in systemic circulation during the acute phase, or its effect may be more pronounced at the intracellular level. In fact, the current literature reports that GSK-3β plays a role as a modulator molecule interacting with Nrf2, NF-κB and inflammatory pathways rather than a direct biomarker in acute ischemic injury [75]. It is assessed that GSK-3β may contribute to the pathophysiological process in acute cerebral ischemia through secondary and indirect effects, but clinical serum levels may be limited in reflecting disease severity [76].

## 6. Limitations

The single-center design and relatively small and limited sample size constitute one of our limitations in terms of generalizability of the findings. Second, biomarker measurements were performed only on serum samples taken within the first 24 h after symptom onset; therefore, temporal changes in oxidative stress and antioxidant responses during disease progression could not be assessed. Third, the cross-sectional nature of the study prevents the establishment of causal relationships between changes in the Nrf2-Keap1 pathway and ischemic damage. Fourthly, another limitation is that clinical stroke severity scales, such as the National Institutes of Health Stroke Scale (NIHSS), were not included in the analysis. Therefore, the relationship between biomarker levels and clinical disease severity could not be evaluated in detail. In addition, adjustments for comorbidities, concomitant medications, and acute-phase treatments could not be comprehensively performed. These factors may influence circulating biomarker concentrations and should be considered as potential confounders. Furthermore, serum biomarker levels may not fully reflect intracellular or tissue-specific molecular dynamics in the brain. Since the measurements were performed in peripheral blood samples, the observed changes should be interpreted as systemic biochemical alterations rather than direct indicators of molecular processes occurring in cerebral tissue. Finally, although high AUC values were observed for some biomarkers, these findings were not validated in an independent cohort. Therefore, the ROC-derived diagnostic performance should be interpreted cautiously and considered exploratory. Larger, multicenter studies with independent validation cohorts are needed to confirm the clinical utility of these biomarkers.

## 7. Conclusions

The present study demonstrates significant alterations in oxidative stress- and antioxidant defense-related biomarkers in patients with acute ischemic stroke. Decreased serum Nrf2 levels together with increased Keap1 levels suggest dysregulation of the Nrf2–Keap1 antioxidant pathway, while elevated 4-HNE levels are consistent with enhanced lipid peroxidation during acute cerebral ischemia. Although increased HO-1 and GSH levels may reflect compensatory antioxidant responses, these changes appear insufficient to fully counterbalance the increased oxidative burden. The absence of a significant difference in circulating GSK-3β levels suggests that its role may not be adequately reflected in serum or may depend on temporal or tissue-specific regulation. Overall, these findings support the presence of altered oxidative stress pathways in acute ischemic stroke. However, the clinical relevance of these biomarkers should be interpreted cautiously, and larger prospective, multicenter studies with independent validation are required before their potential diagnostic or therapeutic value can be established.

## Figures and Tables

**Table 1:** Statistical Analysis of Measured Parameters.

	Patient Group	Control Group	
	Mean ± SD	Mean ± SD	*p* ^a^
Nrf2 (ng/mL)	10.1 ± 3.38	16.31 ± 4.32	0.001 **
Keap 1 (ng/L)	504.01 ± 246.01	345.87 ± 130.23	0.001 **
GSK-3ß (ng/L)	328.87 ± 128.15	298.84 ± 67.34	0.952
HO-1 (ng/mL)	2.49 ± 1.58	2.08 ± 1	0.038 *
GSH (ng/mL)	11.58 ± 4.39	9.82 ± 3.73	0.006 *
4-HNE (ng/L)	350.06 ± 96.32	187.64 ± 59.44	0.001 **

^a^ Mann–Whitney U Test, * *p* < 0.05, ** *p* < 0.001, Patient Group (n = 88), Control Group (n = 72).

## Data Availability

The datasets used during the current study are available from the corresponding author upon request.

## References

[1] Krishnamurthy H.K., Pereira M., Rajavelu I., Jayaraman V., Krishna K., Wang T., Bei K., Rajasekaran J.J. (2024). Oxidative stress: Fundamentals and advances in quantification techniques. Front. Chem..

[2] Taysi S., Tascan A.S., Ugur M.G., Demir M. (2019). Radicals, oxidative/nitrosative stress and preeclampsia. Mini Rev. Med. Chem..

[3] Celik E., Taysi S., Sucu S., Ulusal H., Sevincler E., Celik A. (2019). Urotensin 2 and Oxidative Stress Levels in Maternal Serum in Pregnancies Complicated by Intrauterine Growth Restriction. Medicina.

[4] Taysi M.E., Demirel M.E., Cetinkaya A., Saylan A., Kayis S.A., Alisik M. (2025). Investigation of the effect of quercetin on experimental traumatic cardiac injury in rats. Front. Cardiovasc. Med..

[5] Uygun H., Çiçek Z.N., Ercan K., Taysi S. (2025). A Novel Therapeutic Target for Pediatric Pneumonia: Sestrin2. Medicina.

[6] Ayna A., Caglayan C., Taysi S. (2025). Cellular and Molecular Mechanisms of Oxidative DNA Damage and Repair. Medicina.

[7] Aksoy H., Taysi S., Altinkaynak K., Bakan E., Bakan N., Kumtepe Y. (2003). Antioxidant potential and transferrin, ceruloplasmin, and lipid peroxidation levels in women with preeclampsia. J. Investig. Med..

[8] Koçtürk F., Emekli F., Egi K., Taysi S. (2025). Evaluation of Nrf2/Keap1 Pathway in Patients with Migraine. Medicina.

[9] Uslu C., Taysi S., Bakan N. (2003). Lipid peroxidation and antioxidant enzyme activities in experimental maxillary sinusitis. Ann. Clin. Lab. Sci..

[10] Gökdeniz H.G., Bayramoglu N.T., Taysi S. (2024). Investigation of Nrf2-Keap-1 pathway, Sestrin 2 and oxidative stress markers in serum of patients with placenta Accreata spectrum. Eur. J. Obstet. Gynecol. Reprod. Biol..

[11] Kh Abdulrahman Abdulrahman Z., Inco H., Ercan K., Aytac I., Taysi S. (2025). Investigation of sestrin-2 levels and thiol-disulfide homeostasis in polyp tissue of patients with nasal polyps. Sci. Rep..

[12] Farina M., Vieira L.E., Buttari B., Profumo E., Saso L. (2021). The Nrf2 pathway in ischemic stroke: A review. Molecules.

[13] Feigin V.L., Brainin M., Norrving B., Martins S.O., Pandian J., Lindsay P., Grupper M.F., Rautalin I. (2025). World stroke organization: Global stroke fact sheet 2025. Int. J. Stroke.

[14] He Q., Wang W., Zhang Y., Xiong Y., Tao C., Ma L., Ma J., You C., Wang C. (2024). Global, regional, and national burden of stroke, 1990–2021: A systematic analysis for global burden of disease 2021. Stroke.

[15] Feigin V.L., Owolabi M.O., Abd-Allah F., Akinyemi R.O., Bhattacharjee N.V., Brainin M., Cao J., Caso V., Dalton B., Davis A. (2023). Pragmatic solutions to reduce the global burden of stroke: A World Stroke Organization–Lancet Neurology Commission. Lancet Neurol..

[16] Capone F., Salati S., Vincenzi F., Liberti M., Aicardi G., Apollonio F., Varani K., Cadossi R., Di Lazzaro V. (2022). Pulsed electromagnetic fields: A novel attractive therapeutic opportunity for neuroprotection after acute cerebral ischemia. Neuromodul. Technol. Neural Interface.

[17] Lucas-Noll J., Clua-Espuny J.L., Lleixa-Fortuno M., Gavalda-Espelta E., Queralt-Tomas L., Panisello-Tafalla A., Carles-Lavila M. (2023). The costs associated with stroke care continuum: A systematic review. Health Econ. Rev..

[18] Luengo-Fernandez R., Walli-Attaei M., Gray A., Torbica A., Maggioni A.P., Huculeci R., Bairami F., Aboyans V., Timmis A.D., Vardas P. (2023). Economic burden of cardiovascular diseases in the European Union: A population-based cost study. Eur. Heart J..

[19] Salaudeen M.A., Bello N., Danraka R.N., Ammani M.L. (2024). Understanding the pathophysiology of ischemic stroke: The basis of current therapies and opportunity for new ones. Biomolecules.

[20] Kovacheva E., Gevezova M., Koeva M., Mihaylova V., Kormova V., Kostadinova E., Kostadinova Y., Kazakova M., Sarafian V. (2025). Mitochondrial Function, Oxidative Stress, Inflammation and Thrombolytic Treatment in Ischemic Stroke. Int. J. Mol. Sci..

[21] Li Q., Gao S. (2017). Mitochondrial dysfunction in ischemic stroke. Translational Research in Stroke.

[22] Yang S.-H., Liu R. (2021). Four decades of ischemic penumbra and its implication for ischemic stroke. Transl. Stroke Res..

[23] Bersano A., Gatti L. (2023). Pathophysiology and treatment of stroke: Present status and future perspectives. Int. J. Mol. Sci..

[24] Vos E., Geraedts V., Van Der Lugt A., Dippel D., Wermer M., Hofmeijer J., van Es A., Roos Y., Peeters-Scholte C., van den Wijngaard I. (2022). Systematic review-combining neuroprotection with reperfusion in acute ischemic stroke. Front. Neurol..

[25] Chen W., Teng X., Ding H., Xie Z., Cheng P., Liu Z., Feng T., Zhang X., Huang W., Geng D. (2022). Nrf2 protects against cerebral ischemia-reperfusion injury by suppressing programmed necrosis and inflammatory signaling pathways. Ann. Transl. Med..

[26] Kuo P.-C., Weng W.-T., Scofield B.A., Paraiso H.C., Yu I.-C.I., Yen J.-H.J. (2024). Ischemia-induced endogenous Nrf2/HO-1 axis activation modulates microglial polarization and restrains ischemic brain injury. Front. Immunol..

[27] Zhang X., Wu Q., Wang Z., Li H., Dai J. (2022). Keap1-Nrf2/ARE signal pathway activated by butylphthalide in the treatment of ischemic stroke. Am. J. Transl. Res..

[28] Soni D., Kumar P. (2022). GSK-3β-mediated regulation of Nrf2/HO-1 signaling as a new therapeutic approach in the treatment of movement disorders. Pharmacol. Rep..

[29] Altunbas G., Kaplan M., Duzen V., Kaya E.E., Gokdeniz H.G., Taysi S. (2024). Determination of Serum Glycogen Synthase 3 Beta Levels in Patients with Heart Failure, a Novel Marker for Diagnosis and Defining Disease Severity?. Arq. Bras. Cardiol..

[30] O’Rourke S.A., Shanley L.C., Dunne A. (2024). The Nrf2-HO-1 system and inflammaging. Front. Immunol..

[31] Lane D.J., Alves F., Ayton S.J., Bush A.I. (2023). Striking a NRF2: The rusty and rancid vulnerabilities toward ferroptosis in Alzheimer’s disease. Antioxid. Redox Signal..

[32] Milkovic L., Zarkovic N., Marusic Z., Zarkovic K., Jaganjac M. (2023). The 4-hydroxynonenal–protein adducts and their biological relevance: Are some proteins preferred targets?. Antioxidants.

[33] Werring D., Adams M., Benjamin L., Brown M., Chandratheva A., Cowley P., Grieve J., Humphries F., Jäger H.R., Losseff N. (2024). Stroke and Cerebrovascular Diseases. Neurology: A Queen Square Textbook.

[34] Zhang M., Liu Q., Meng H., Duan H., Liu X., Wu J., Gao F., Wang S., Tan R., Yuan J. (2024). Ischemia-reperfusion injury: Molecular mechanisms and therapeutic targets. Signal Transduct. Target. Ther..

[35] Feng S., Yang M., Liu S., He Y., Deng S., Gong Y. (2023). Oxidative stress as a bridge between age and stroke: A narrative review. J. Intensive Med..

[36] Allen C.L., Bayraktutan U. (2009). Oxidative stress and its role in the pathogenesis of ischaemic stroke. Int. J. Stroke.

[37] Kaspar J.W., Niture S.K., Jaiswal A.K. (2009). Nrf2: INrf2 (Keap1) signaling in oxidative stress. Free Radic. Biol. Med..

[38] Ma Q. (2013). Role of nrf2 in oxidative stress and toxicity. Annu. Rev. Pharmacol. Toxicol..

[39] Zhao X., Sun G., Zhang J., Strong R., Dash P.K., Kan Y.W., Grotta J.C., Aronowski J. (2007). Transcription factor Nrf2 protects the brain from damage produced by intracerebral hemorrhage. Stroke.

[40] Zhang M., An C., Gao Y., Leak R.K., Chen J., Zhang F. (2013). Emerging roles of Nrf2 and phase II antioxidant enzymes in neuroprotection. Prog. Neurobiol..

[41] Wang L., Zhang X., Xiong X., Zhu H., Chen R., Zhang S., Chen G., Jian Z. (2022). Nrf2 regulates oxidative stress and its role in cerebral ischemic stroke. Antioxidants.

[42] Iadecola C., Anrather J. (2011). The immunology of stroke: From mechanisms to translation. Nat. Med..

[43] Lambertsen K.L., Finsen B., Clausen B.H. (2019). Post-stroke inflammation—Target or tool for therapy?. Acta Neuropathol..

[44] Houldsworth A. (2024). Role of oxidative stress in neurodegenerative disorders: A review of reactive oxygen species and prevention by antioxidants. Brain Commun..

[45] Rodrigo R., Fernandez-Gajardo R., Gutiérrez R., Manuel Matamala J., Carrasco R., Miranda-Merchak A., Feuerhake W. (2013). Oxidative stress and pathophysiology of ischemic stroke: Novel therapeutic opportunities. CNS Neurol. Disord.-Drug Targets (Former. Curr. Drug Targets-CNS Neurol. Disord.).

[46] Cherubini A., Ruggiero C., Polidori M.C., Mecocci P. (2005). Potential markers of oxidative stress in stroke. Free Radic. Biol. Med..

[47] Chen H., Yoshioka H., Kim G.S., Jung J.E., Okami N., Sakata H., Maier C.M., Narasimhan P., Goeders C.E., Chan P.H. (2011). Oxidative stress in ischemic brain damage: Mechanisms of cell death and potential molecular targets for neuroprotection. Antioxid. Redox Signal..

[48] Kensler T.W., Wakabayashi N., Biswal S. (2007). Cell survival responses to environmental stresses via the Keap1-Nrf2-ARE pathway. Annu. Rev. Pharmacol. Toxicol..

[49] Loboda A., Damulewicz M., Pyza E., Jozkowicz A., Dulak J. (2016). Role of Nrf2/HO-1 system in development, oxidative stress response and diseases: An evolutionarily conserved mechanism. Cell. Mol. Life Sci..

[50] Zhang D.D. (2006). Mechanistic studies of the Nrf2-Keap1 signaling pathway. Drug Metab. Rev..

[51] Dodson M., Castro-Portuguez R., Zhang D.D. (2019). NRF2 plays a critical role in mitigating lipid peroxidation and ferroptosis. Redox Biol..

[52] Jin W., Wang H., Yan W., Zhu L., Hu Z., Ding Y., Tang K. (2009). Role of Nrf2 in protection against traumatic brain injury in mice. J. Neurotrauma.

[53] Cuadrado A., Manda G., Hassan A., Alcaraz M.J., Barbas C., Daiber A., Ghezzi P., León R., López M.G., Oliva B. (2018). Transcription factor NRF2 as a therapeutic target for chronic diseases: A systems medicine approach. Pharmacol. Rev..

[54] Alfieri A., Srivastava S., Siow R.C., Modo M., Fraser P.A., Mann G.E. (2011). Targeting the Nrf2–Keap1 antioxidant defence pathway for neurovascular protection in stroke. J. Physiol..

[55] Tian C., Gao L., Zucker I.H. (2021). Regulation of Nrf2 signaling pathway in heart failure: Role of extracellular vesicles and non-coding RNAs. Free Radic. Biol. Med..

[56] Yagishita Y., Gatbonton-Schwager T.N., McCallum M.L., Kensler T.W. (2020). Current landscape of NRF2 biomarkers in clinical trials. Antioxidants.

[57] Suzuki T., Takahashi J., Yamamoto M. (2023). Molecular basis of the KEAP1-NRF2 signaling pathway. Mol. Cells.

[58] Suzuki T., Yamamoto M. (2015). Molecular basis of the Keap1–Nrf2 system. Free Radic. Biol. Med..

[59] Liu L., Locascio L.M., Doré S. (2019). Critical role of Nrf2 in experimental ischemic stroke. Front. Pharmacol..

[60] Uchida K. (2003). 4-Hydroxy-2-nonenal: A product and mediator of oxidative stress. Prog. Lipid Res..

[61] Jazwa A., Cuadrado A. (2010). Targeting heme oxygenase-1 for neuroprotection and neuroinflammation in neurodegenerative diseases. Curr. Drug Targets.

[62] Hannan M.A., Dash R., Sohag A.A.M., Haque M.N., Moon I.S. (2020). Neuroprotection against oxidative stress: Phytochemicals targeting TrkB signaling and the Nrf2-ARE antioxidant system. Front. Mol. Neurosci..

[63] Bono S., Feligioni M., Corbo M. (2021). Impaired antioxidant KEAP1-NRF2 system in amyotrophic lateral sclerosis: NRF2 activation as a potential therapeutic strategy. Mol. Neurodegener..

[64] Chamorro Á., Lo E.H., Renú A., van Leyen K., Lyden P.D. (2021). The future of neuroprotection in stroke. J. Neurol. Neurosurg. Psychiatry.

[65] Wu L., Xiong X., Wu X., Ye Y., Jian Z., Zhi Z., Gu L. (2020). Targeting oxidative stress and inflammation to prevent ischemia-reperfusion injury. Front. Mol. Neurosci..

[66] Ayala A., Muñoz M.F., Argüelles S. (2014). Lipid peroxidation: Production, metabolism, and signaling mechanisms of malondialdehyde and 4-hydroxy-2-nonenal. Oxid. Med. Cell. Longev..

[67] Lee W.-C., Wong H.-Y., Chai Y.-Y., Shi C.-W., Amino N., Kikuchi S., Huang S.-H. (2012). Lipid peroxidation dysregulation in ischemic stroke: Plasma 4-HNE as a potential biomarker?. Biochem. Biophys. Res. Commun..

[68] Guo J.-M., Liu A.-J., Zang P., Dong W.-Z., Ying L., Wang W., Xu P., Song X.-R., Cai J., Zhang S.-Q. (2013). ALDH2 protects against stroke by clearing 4-HNE. Cell Res..

[69] Butterfield D.A., Halliwell B. (2019). Oxidative stress, dysfunctional glucose metabolism and Alzheimer disease. Nat. Rev. Neurosci..

[70] Sweeney M.D., Sagare A.P., Zlokovic B.V. (2018). Blood–brain barrier breakdown in Alzheimer disease and other neurodegenerative disorders. Nat. Rev. Neurol..

[71] Deng X., Chu W., Zhang H., Peng Y. (2023). Nrf2 and ferroptosis: A new research direction for ischemic stroke. Cell. Mol. Neurobiol..

[72] Beurel E., Grieco S.F., Jope R.S. (2015). Glycogen synthase kinase-3 (GSK3): Regulation, actions, and diseases. Pharmacol. Ther..

[73] Sharma R., Kumari S., Kapoor S., Deshmukh R., Singh T.G. (2024). Role of GSK-3 Inhibition in Modulating the Pathology of Stroke. Ischemic Injury.

[74] Medina M., Garrido J.J., Wandosell F.G. (2011). Modulation of GSK-3 as a therapeutic strategy on tau pathologies. Front. Mol. Neurosci..

[75] Cuadrado A., Rojo A.I., Wells G., Hayes J.D., Cousin S.P., Rumsey W.L., Attucks O.C., Franklin S., Levonen A.-L., Kensler T.W. (2019). Therapeutic targeting of the NRF2 and KEAP1 partnership in chronic diseases. Nat. Rev. Drug Discov..

[76] Paspalj D., Nikic P., Savic M., Djuric D., Simanic I., Zivkovic V., Jeremic N., Srejovic I., Jakovljevic V. (2015). Redox status in acute ischemic stroke: Correlation with clinical outcome. Mol. Cell. Biochem..

